# Seafloor hydrothermal activity along mid-ocean ridge with strong melt supply: study from segment 27, southwest Indian ridge

**DOI:** 10.1038/s41598-019-46299-1

**Published:** 2019-07-08

**Authors:** Xihe Yue, Huaiming Li, Jianye Ren, Chunhui Tao, Jianping Zhou, Yuan Wang, Xiaoxia Lü

**Affiliations:** 1 College of Marine Science and Technology, China University of Geosciences, Wuhan, 430074 China; 2 Key Laboratory of Submarine Geosciences, State Oceanic Administration & Second Institute of Oceanography, Ministry of Natural Resources, Hangzhou, 310012 China; 3 Institute of Oceanography, Shanghai Jiao Tong University, Shanghai, 200030 China

**Keywords:** Physical oceanography, Geophysics, Geology

## Abstract

Continuous tow investigations have shown that the present vent field inventory along fast to intermediate spreading ridges may be underestimated by at least 3–6 times, while the limited towed line investigations of venting sites along slow to ultra-slow spreading ridges make it impossible to determine their distribution. The Chinese Dayang cruise has conducted detailed towed line surveys of hydrothermal activity on segment 27 of the ultra-slow spreading southwest Indian ridge in 2015. The results have identified as many as 9 hydrothermal fields along 85-km-long segment, including one confirmed hydrothermal field, three inferred hydrothermal fields and five suspected fields. Hydrothermal activities are not only limited along-axis but also found approximately 10 km away from the axis. These vent fields are likely powered by a seismically identified axial magma chamber, including melt migration along normal faults to flank areas. The calculated hydrothermal activity frequency on segment 27 is approximately 3.6–8 times higher than that calculated from the Interridge database, suggesting that careful system exploration can reveal more hydrothermal activities even on ultra-slow spreading ridges effected by hotspot.

## Introduction

Hydrothermal activity is widespread on the seafloor, such as at ocean spreading ridges (OSRs) and arc volcanoes^1^. The study of hydrothermal field plays a key role in ocean-crust interactions, the formation of seafloor massive sulfide deposits and oceanic chemical and biogeochemical cycles^2–5^. Approximately 700 hydrothermal fields have been discovered worldwide, and OSRs hydrothermal activity comprises 75% of the total (data from www.vents-data.interridge.org/). However, approximately ~70% of the 71,000 km OSRs still have had no investigations of their hydrothermal activity^2,5,6^.

Hydrothermal activity distribution on the OSRs is expressed by the fraction of the ridge crest length overlain by a significant hydrothermal plume (Ph) or the vent site frequency Fs (sites per 100 km of ridge length) and is primarily controlled by the magmatic budget^1^. The Ph and Fs have positive linear correlations with the ridge spreading rate^1,5^. In recent studies, several fast to intermediate ridges have been surveyed by continuous tows with multiple sensors that can detect both particle-rich and particle-poor plumes. Scientists have found that the present Fs along that  spreading ridges may be underestimated by at least 3–6 times^3^. Whether similar distribution frequency is underestimated on slow to ultra-slow spreading ridges is not clear because most surveys on these ridges have been vertical profiles and continuous tow instruments have been rarely used^7–9^. Our research on the southwest Indian ridge (SWIR) provides more detailed evidence about the global vent fields distribution.

One interesting subset of mid-ocean ridges (MORs) includes the ridges affected by hotspots, which comprise 15–20% of the total length of the MORs^10–12^. Ridges affected by hotspots have high magma supply, thickened crust and have less hydrothermal activity than normal MORs^10,13^. For example, at the slow-spreading south Atlantic Ridge at 7°–11°S near the Ascension hotspot the shallowest location has crustal thicknesses up to 11 km^9,14^, and only one diffuse hydrothermal field^15^ and one high-temperature hydrothermal field have been identified^16^. Surveys using Conductivity–Temperature–Depth (CTD) sensors have obtained 175 vertical profiles along the Reykjanes Ridge (57°45′–63°09′N), which is above the Iceland hotspot, and identified only one hydrothermal field at 63.1°N (named Steinahóll)^17^. However, our survey of hydrothermal activity on the ultra-slow SWIR which interacts with the Crozet hotspot shows obvious differences from previous studies. Whether or not the hydrothermal activity is suppressed on the hotspot-affected MORs needs to be investigated^10,18^.

**Figure 1:**
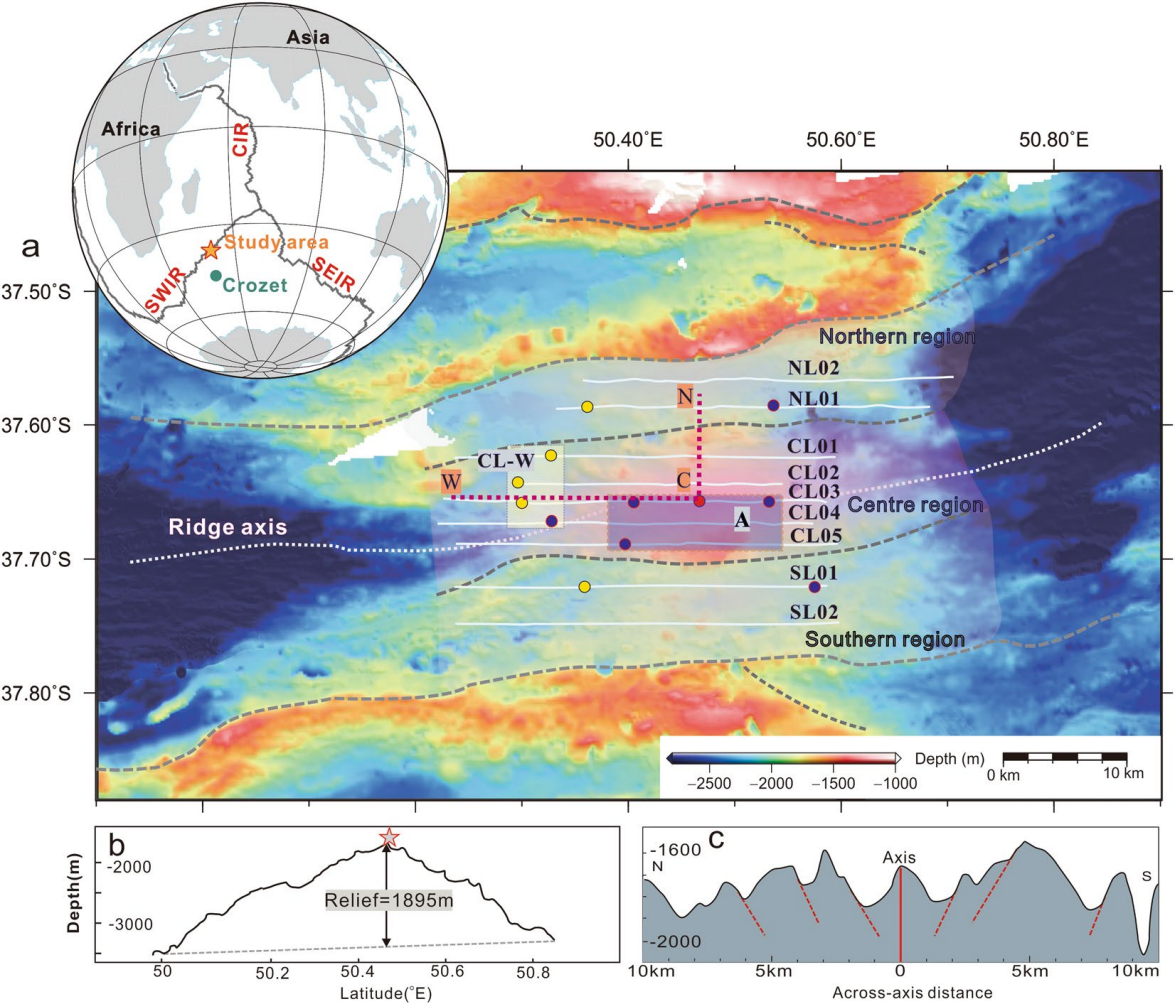
Geological background of segment 27 on the SWIR (Created by the Generic Mapping Tools (GMT version 5), from http://gmt.soest.hawaii.edu/. The topography is from multibeam sonar data by Chinese *Dayang* cruises). (**a**) Location map of segment 27 on the SWIR. The white lines show the DHDS survey lines of the 34^th^ *Dayang* cruise. The white dotted lines indicate the axial volcanic ridge (AVR). The red circle denotes the Duanqiao hydrothermal field (DQF). The yellow circles mark the locations of inferred hydrothermal fields and the dark blue circles are the locations of suspected hydrothermal fields. The red dotted line CN marks the location of the vertical cross-section across the segment centre in the spreading direction, which is shown in Fig. 7, and line CW is the cross-section along the ridge axis, which is also shown in Fig. 7. The gray dashed curves are bounding faults^23^. The left inset shows the location of the study area (Created by the Generic Mapping Tools (GMT version 5). Ridge data from http://www-udc.ig.utexas.edu). SWIR: Southwest Indian Ridge; CIR: Central Indian Ridge; SEIR: Southeast Indian Ridge. (**b**) Bathymetric along-axis section at the centre of segment 27. (**c**) Bathymetric cross-section at the centre of segment 27.

Our study area is located between the Indomed and Gallieni transform faults (In-Ga TFs), on an ultra-slow spreading ridge with a spreading rate of 11.9 mm/yr. The area is one of the shallowest regions of the SWIR with a mean axial depth of 3180 m^19^. Segment 27 is 85 km long and has along-axis relief of 1895 m^19,20^. And the presence of off-axis dome volcanoes that reflect a long-term magma supply^21^. The seafloor quickly deepens away from the volcano along the ridge axis, bounded by two ridgeward-dipping bounding fault scarps defining a 20 to 25-km-wide median valley(Fig. 1)^22^. According to the location of the bounding fault, we divided the study area into 3 parts: centre region, southern region and northern region (Fig. 1). Mantle Bouguer anomaly (MBA) values of segment 27 decrease to a low of −84 mGal, indicating a thick crust and/or hot mantle along the axis^19^. Seismic tomography and full waveform inversion of ocean bottom seismometer data indicate the presence of a large low-velocity anomaly (LVA) approximately ~4–9 km below the seafloor interpreted as an axial magma chamber (AMC) in the lower crust^23^. Magmatism in this area is related to the Crozet hotspot, which is located 1000 km south of the ridge axis^19^. The basalts in the study area are enriched in isotopes and trace elements compared with depleted, normal-MOR basalt (N-MORB) which is considered geochemical evidence of interaction between the Crozet hotspot and the ridge^24^. The Duanqiao hydrothermal field (DQF) (50.47°E) was discovered on the central axis ridge of segment 27 in 2007 by the Chinese *Dayang* cruise. The hydrothermal field has an area of approximately 200 × 125 m^25^.

In 2015, 34^th^ Chinese *Dayang* cruise conducted systematic surveys of hydrothermal activity on segment 27 using a deep-towed hydrothermal detection system (DHDS) and obtained both optical and chemical data. Nine survey lines at spacing of 0.8–3 km, approximately 390 km in total length, surveyed an area of nearly 1300 km^2^.

Hydrothermal plumes are rich in metallic elements and suspended particles and higher temperatures than the surrounding seawater. In addition, hydrothermal altered rocks, sediments (called “seabed types anomaly” in this paper), and fauna around vents differ greatly from the normal seafloor. Physical and chemical evidence in the water column such as turbidity, oxidation-reduction potential (ORP), temperature, dissolved Mn, CH_4_, Fe and ^3^He, seabed types and hydrothermal vent fauna are often used to determine the locations of hydrothermal fields^15,26^ (Sup. Table 1).

**Figure 2:**
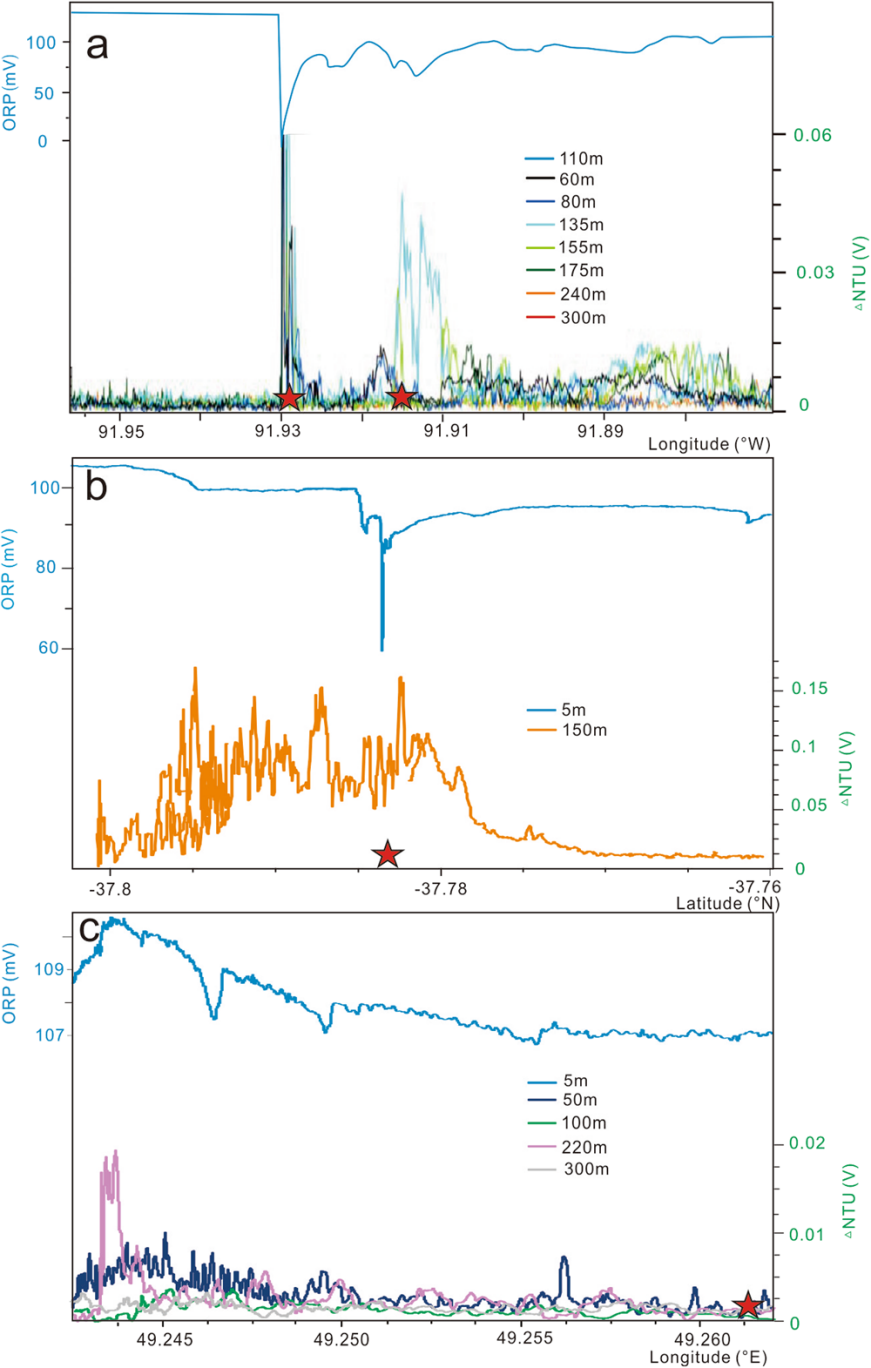
Typical profiles of different hydrothermal fields. (**a**,**b**), and (**c**) show the distributions of ORP and NTU anomalies at a different altitude. The red stars are the locations of hydrothermal fields. (**a**) Iguanas-Pinguinos hydrothermal field on the GSC^10^; (**b**) Longqi-1 hydrothermal field on the SWIR; (**c**) Yuhuang-1 hydrothermal field on the SWIR.

One common method of identifying hydrothermal activity is combining anomalies from sensors and visual images. NTU which is turbidity anomaly detected from Miniature Autonomous Plume Recorders (MAPRs), oxidation-reduction potential (ORP) or dE/dt(the value of the time derivative of ORP) which is the anomaly decrease read from ORP sensor and seabed anomaly are three indices using to identify hydrothermal activity^3^. For example, Iguanas-Pinguinos hydrothermal field on the Gala’pagos Spreading Center (GSC) which has high-temperature “black smokers”, was surveyed by towed lines. The plumes extend for approximately 11 km and rise height approximately 100 m with ΔNTU of >0.06 and a decrease in ORP of approximately 100 mV (Fig. 2a)^10^. During the 34^th^ Chinese *Dayang* cruise, towed lines were performed using the same detection technology and method along the 49–52°E, SWIR, including three second-order segments called segment 27, 28 and 29. Lines passed through confirmed hydrothermal fields, such as the Longqi hydrothermal field (LHF, 49.65°E 37.782°S) on segment 28 (Sup. Fig. 1), and the Yuhuang hydrothermal field (YHF, 49.263°E -37.938°S) on segment 29 (Sup. Fig. 1). Previous study indicated that LHF was an area of focused hydrothermal venting^25^. The YHF is mostly inactive and is covered by sediment^27^. Obvious differences in the NTU and ORP anomalies between LHF and YHF are shown in the profiles (Fig. 2). Similar to the Iguanas-Pinguinos hydrothermal field, the LHF has a plume that extends for approximately 2 km and rises to a height of 150 m with ΔNTU > 0.1 and an ORP decrease of approximately 40 mV (Fig. 2b). Focused-flow hydrothermal vents have not been discovered on the YHF where a weak NTU anomaly (ΔNTU = 0.01–0.02), a decrease in ORP of approximately 2 mV and a plume height of approximately 50–100 m (Fig. 2c) were identified near the site.

## Results

During our study, we use NTU and ORP anomaly which is summarized from other hydrothermal vents’ anomaly values (Sup. Table 1) to identify the hydrothermal site in segment 27. In this paper, an NTU anomaly is a plume spread more than 1 km and rise height above 100 m with the value above 0.01NTU, and an ORP anomaly is identified by a dE/dt value more negative than −0.04 mV/s for consecutive measurements with an overall decrease (dE) >2 mV^3^. According to the limited scale of the video and camera, seabed type shows no anomaly in the study area. We categorized the hydrothermal fields into inferred fields and suspected fields: the inferred fields have ORP and NTU anomalies to avoid the influence of resuspended bottom sediments^8^ and ORP sensor instability; the suspected fields have only one index such as NTU anomaly, or a decrease in ORP. Base on the analysis of the NTU and ORP data, the combinations of 7 NTU and 10 ORP anomalies identified as many as 9 distinct fields. The hydrothermal fields on segment 27 include one confirmed hydrothermal field (DQF), three inferred hydrothermal fields (i.e., NL01, SL01, and CL-W) and five suspected fields (i.e., CL03-2, CL03-3, CL05, NL01-2, and SL01-2) (Fig. 1) (Sup. Table 2 provides detailed information about each site). The DQF was confirmed through geological sampling; it had no NTU anomaly and a decrease in ORP (dE/dt) of approximately 10 mV (0.1–0.2 mV/s). The seabed type at DQF is mainly basalt and metalliferous sediments. The three inferred hydrothermal fields all have NTU anomalies (ΔNTU = 0.01–0.04) and decreases in ORP (dE/dt) between 3 and 25 mV (0.05–0.3 mv/s) with plume heights greater than 200 m. The five suspected sites were detected by obvious decreases in ORP (dE/dt) between 3 and 19 mV (0.12–0.29 mv/s) or NTU anomalies (ΔNTU = 0.01–0.02). Some ORP-only anomaly sites were excluded where videos recorded the towed body hitting the seafloor, this may cause incorrect records of the ORP sensor, such as the ORP decrease in CL04 at 50.43°E and ORP decrease in NL02 at 50.37°E.

**Figure 3:**
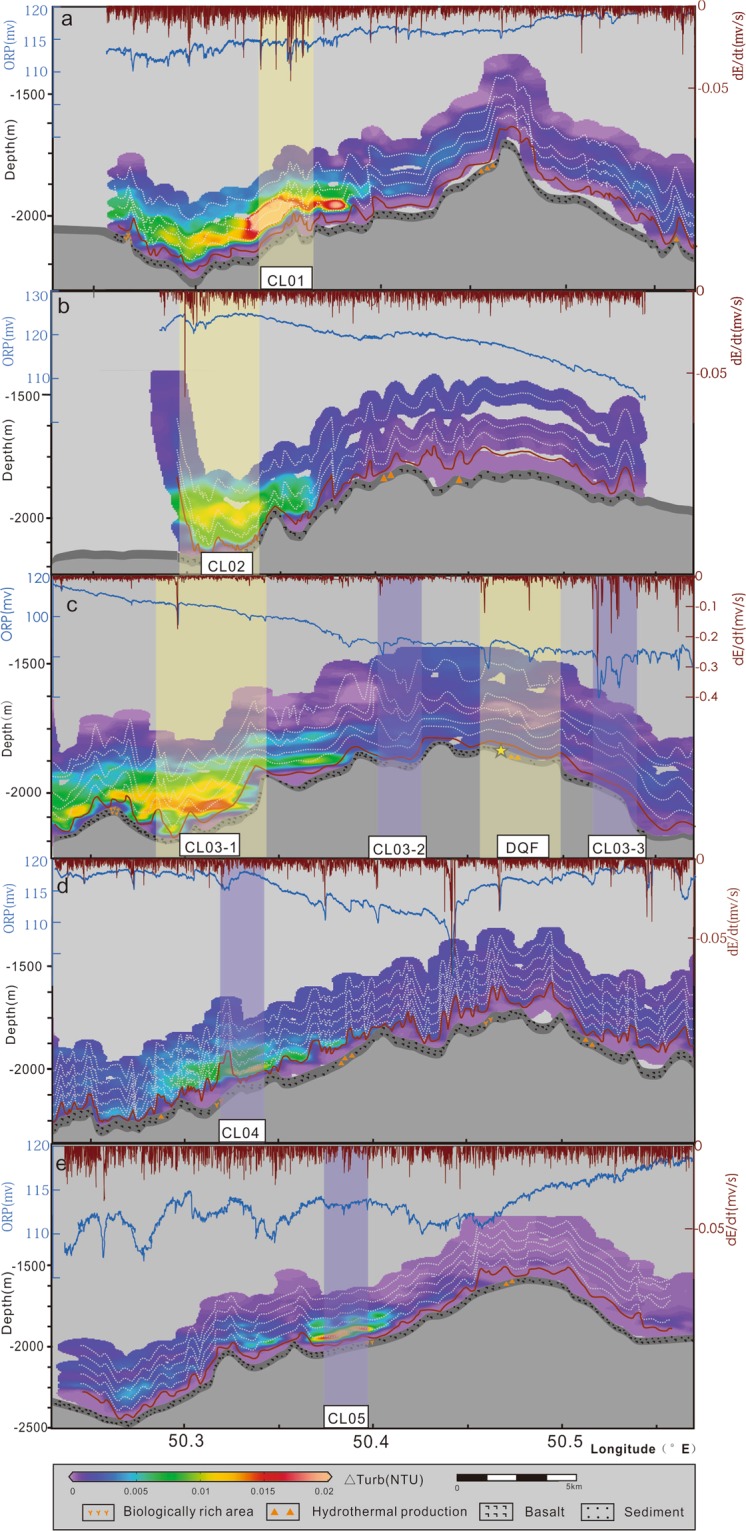
Hydrothermal plume distributions revealed by centre region DHDS lines. (**a**), (**b**), (**c**), (**d**), and (**e**) show the relief, seabed type, NTU, ORP, and negative dE/dt distributions along the CL01 line, CL02 line, CL03 line, CL04 line, and CL05 line, respectively. The top blue line is ORP and the top brown line is dE/dt. The white dotted lines are the traces of MAPRs and the brown lines on the bottom are the traces of the ORP sensor. The yellow bands indicate inferred hydrothermal sites, and the purple bands indicate hydrothermal suspected sites. The grid NTU is created by Ocean Data View (ODV) (Schlitzer, R., Ocean Data View, odv.awi.de, 2018).

Five DHDS survey lines covered the centre region of segment 27 (Fig. 1). Base on the mapping of the plume, five hydrothermal fields (i.e. DQF, CL03-2, CL03-3, CL05, and CL-W) were found in the central axial valley of segment 27 (Figs 1 and 3). Along the central part of the ridge, area A (light violet rectangle area in Fig. 1) contains the DQF and three suspected fields (CL03-2, CL03-3, and CL05). Fields CL03-2 and CL03-3 are located approximately 5–10 km to the east and west of the DQF, respectively, with decreases in ORP (dE/dt) of −0.1 and −0.3, respectively. The two fields have similar decreases in ORP (dE/dt) to those reported in the DQF (dE/dt = −0.2) and no NTU anomalies (Fig. 3c). The CL05 plume has an ΔNTU value of approximately 0.04 and is restricted to 50 m above the seafloor; therefore, we cannot rule out the influence of resuspended bottom sediments. In the western area of the rift valley (Fig. 1), one inferred hydrothermal field named CL-W have been found (light yellow rectangle area in Fig. 1) which including four plumes CL01, CL02, CL03-1, and CL04 that have similar rise heights. The CL01 plume extends for more than 10 km. The MAPRs found consistent NTU anomalies of up to 0.03 approximately 100 m above the seafloor. Decreases in ORP (dE/dt) correspond to the NTU anomalies with values of approximately 0.04 mV/s. At CL02, the plume extends continuously for 5 km. Sensors recorded NTU anomalies reaching 0.02 and decreases in ORP of approximately 3 mV. ΔNTU peak was detected on several MAPRs between 50 and 250 m above the seafloor. At CL03-1 plume extends continuously for 9 km. NTU anomalies fill the axial valley from the seafloor to 150 m above the seabed, and the decrease in ORP (dE/dt) is 11.4 (−0.15 mV/s). The CL04 plume has weak ΔNTU values (0.01~0.02) up to 150 m but no decrease in the ORP.

**Figure 4:**
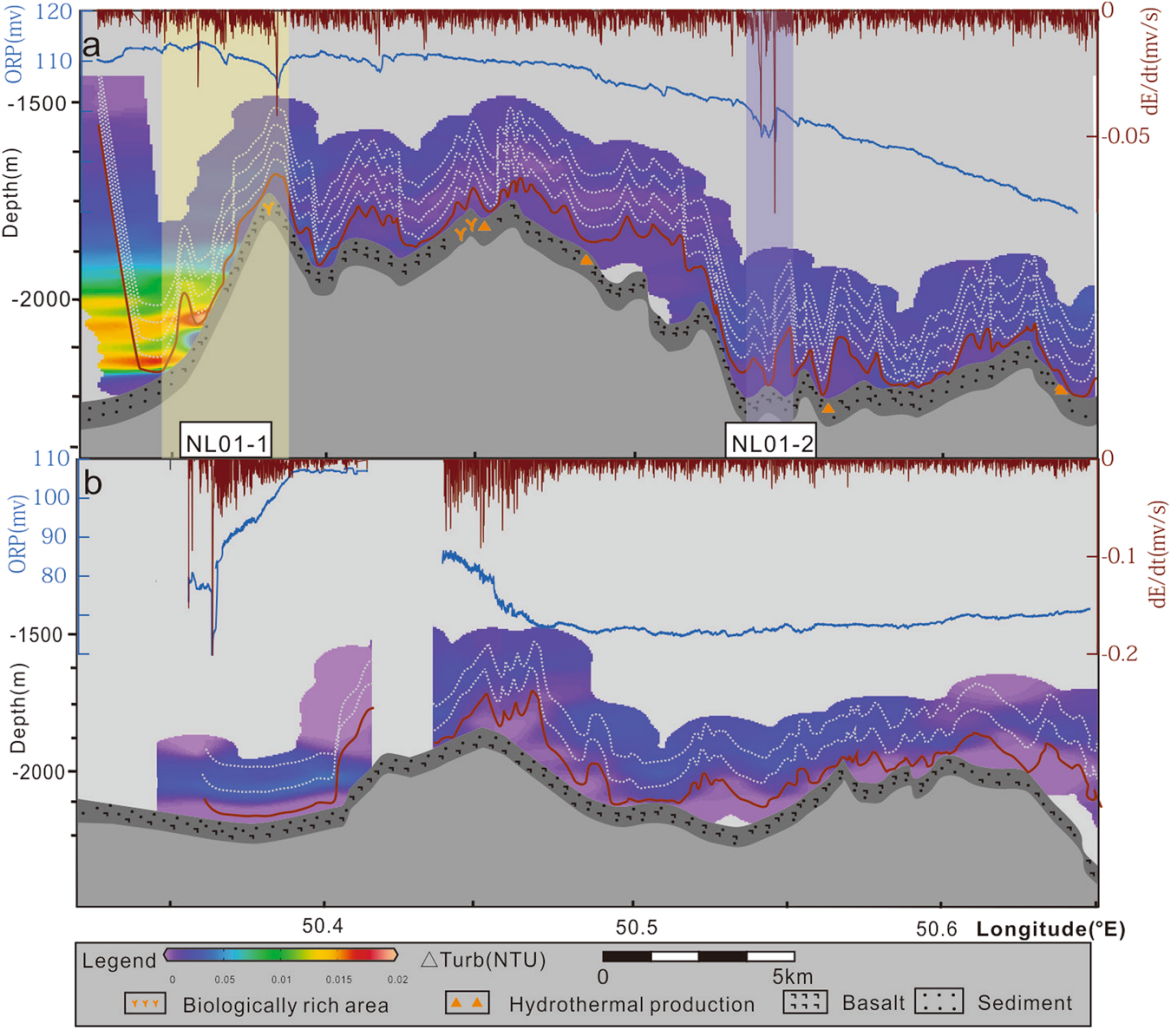
Hydrothermal plume distributions revealed by DHDS lines on the northern region. (**a**) and (**b**) show the relief, seabed type, NTU, ORP and negative dE/dt distributions along NL01line and NL02 line, respectively. The top blue line is ORP and the top brown line is dE/dt. The white dotted lines are the traces of the MAPRs and the brown lines on the bottom are the traces of the ORP sensor. The grid NTU is created by Ocean Data View (ODV) (Schlitzer, R., Ocean Data View, odv.awi.de, 2018).

In the northern region, two lines (NL01 and NL02) were surveyed. Inferred NL01-1 field on the western part of the line is located approximately 10 km from the segment’s centre. NL01-1 has a decrease in ORP (dE/dt = 0.05) and NTU anomaly with a plume height greater than 200 m. NL01-2 is a suspected field that has an ORP decrease of 3.9 mV. No obvious anomalies were detected on line NL02 (Fig. 4).

**Figure 5:**
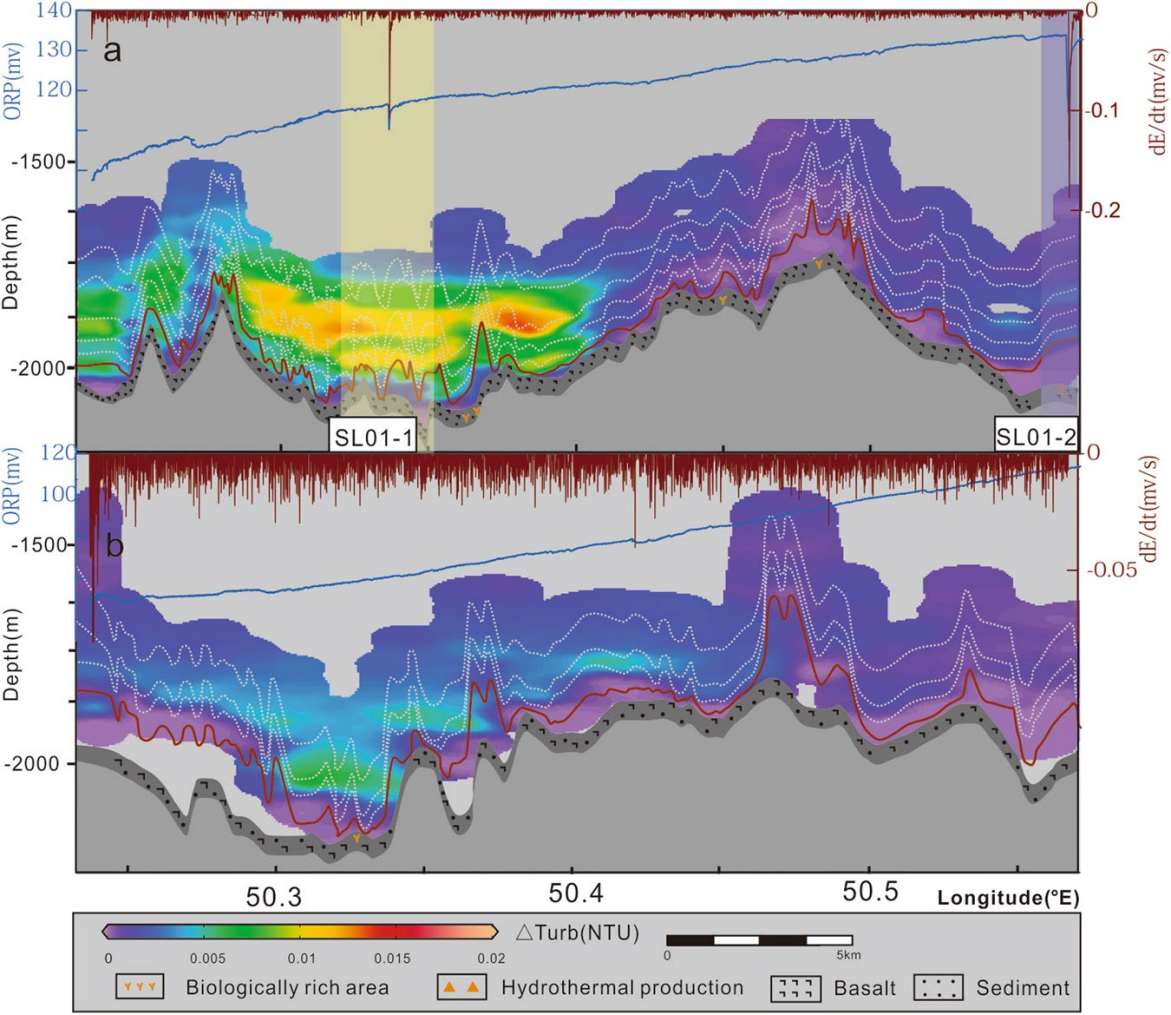
Hydrothermal plume distributions revealed by DHDS lines on the southern region. (**a**,**b**) indicate the relief, seabed type, NTU, ORP, and negative dE/dt distributions along SL01 line and SL02 line, respectively. The top blue line is ORP and the top brown line is dE/dt. The white dotted lines are the traces of MAPRs and the brown lines on the bottom are the traces of the ORP sensor. The grid NTU is created by Ocean Data View (ODV) (Schlitzer, R., Ocean Data View, odv.awi.de, 2018).

The analyses of the NTU and ORP data from two survey lines on the southern region (SL01 and SL02) reveals one inferred field (SL01-1) and one suspected field (SL01-2) (Fig. 5). At SL01-1, the scale of the water column anomaly is approximately 10 km and the ΔNTU exceeds 0.02 at 150–200 m above the seafloor. A decrease in ORP of 6 mV was detected in the plume area. SL01-2 field shows a decrease in ORP of approximately 0.2 mV/s.

## Discussion

### Widespread plumes along segment 27

**Figure 6:**
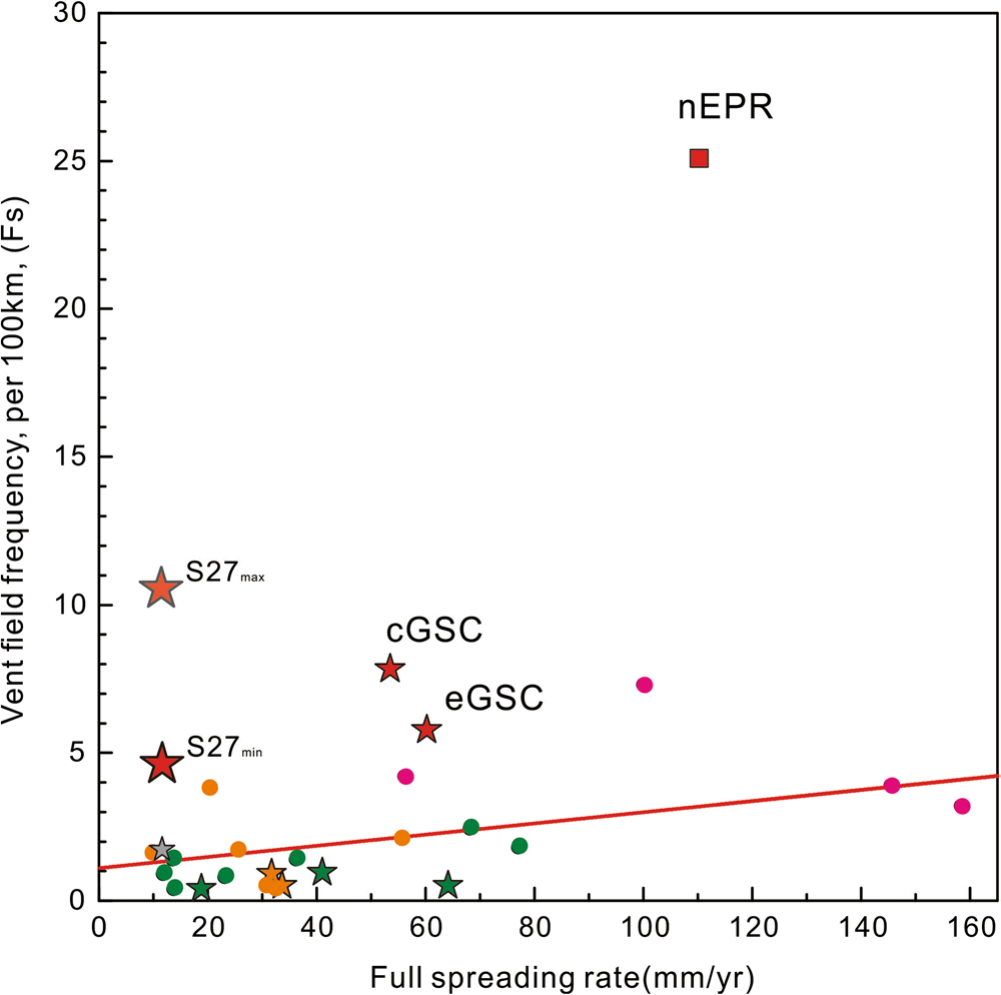
Vent field spatial frequency (Fs) versus full spreading rate for mid-ocean ridge sections. Stars denote ridges influenced by hotspots^1,10^. The magenta circles are MORs with systematic line surveys and the orange circles are ridges with vertical cast stations that have been at least partially studied using ROV/AUV investigations. The green circles are MORs that have only had vertical casts investigations (data from www.vents-data.interridge.org/). The red square and stars indicate MORs with spatially continuous surveys obtained from NTU and ORP sensors^3^. eGSC is the eastern Galápagos Spreading Center (85.7°–91°W)^6^, cGSC is the central Galápagos Spreading Centre (91°–94.9°W)^3^, nEPR is the northern East Pacific Rise (9–10°N)^6^, and S27 is the study area. The red line is fit of vent field frequency to spreading rate^5^. Note: the back-arc spreading centers’ Fs value is not on the graph, discussion is limited to mid-ocean ridges.

For that segment has been basically surveyed, we used the segment measured length (85 km) as the survey length to calculate the vent frequency of segment 27^5^. Considering the area has 5 suspected fields, if we use as many as 9 hydrothermal fields that the calculated Fs_max_ value for segment 27 is 10.6 (light red star in Fig. 6) or the calculated Fs_min_ value is 4.7 (red star in Fig. 6) only considering the inferred and confirmed fields. If we use the interridge database data, only one confirmed field that was discovered before the 34^th^ cruise, the previous Fs value of segment 27 would be 1.3 (gray star in Fig. 6)^1,5^. The calculate Fs value is almost 3.6–8 times higher than the value calculated from the interridge database.

Plume fields with combined optical and ORP anomalies that are >200 m thick, extend for ~3–10 km along the axis and have extensive areas of ΔNTU > 0.015 are considered to be high-temperature plumes, whereas those with only ORP anomalies are likely to be diffuse flow. At least 3 inferred focused fields and 5 suspected diffuse/ORP-only fields have been found here (Sup. Table 2). ORP-only particle-poor vent sites make up approximately 25% of the observed fields on some sections of intermediate to fast spreading ridges^6^ for the high-resolution measurements of closely spaced sources^6^. And our result on segment 27, SWIR found that the ORP-only fields made up as many as half of the fields (5 of 9 fields). Fs values are probably underestimated in ultra-slow spreading ridges for few continuous line surveys have been conducted on that ridges (Fig. 6).

The detailed analyses of the ORP data show that for the eGSC which is affected by the Galapagos hotspot, Fs increases from 1.4 to 6.5 (Fig. 6)^3^. The high-frequency of hydrothermal activity at segment 27 and the GSC indicate that even areas of hotspot-ridge interaction can host more vents than currently expected (Fig. 6). More detailed surveys, such as those on the GSC and segment 27 are needed to confirm that the frequency of hydrothermal activities in hot-spot ridge interaction areas.

### Factors controlling hydrothermal activity along segment 27

**Figure 7:**
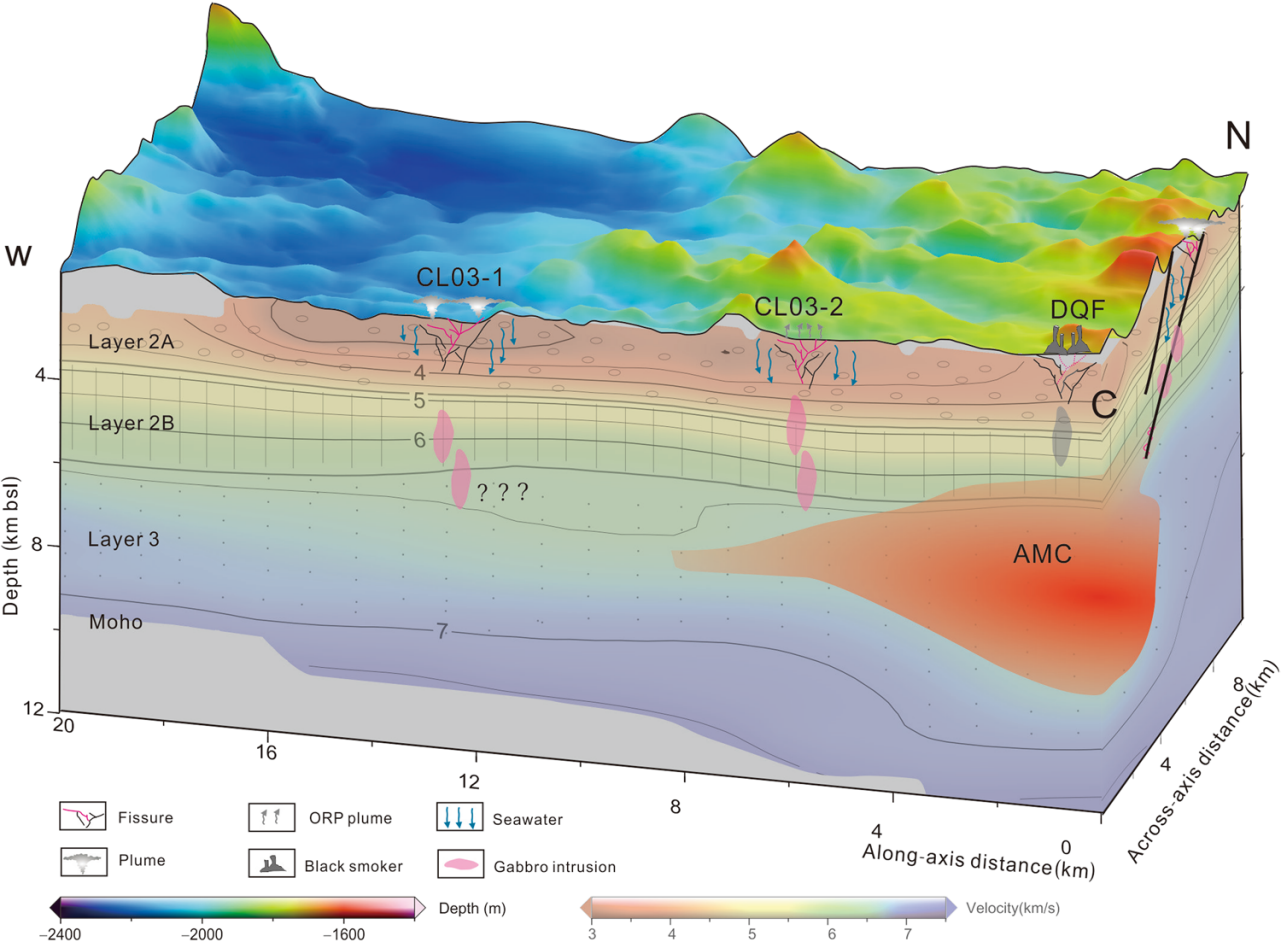
Inferred factors controlling the hydrothermal activity along segment 27. The locations of the sections are marked in Fig. 1. The seismic velocity model data and the location of AMC is from previous studies^22,38^.

Geological and geophysical studies provide information about the structure of the lithosphere, as well as magma melt migration along segment 27 (Fig. 7)^22,28^. The AMC is located ~4–9 km below the seafloor, deeper than AMCs observed at fast-spreading ridges^23^, and is likely the heat source for hydrothermal discharge along segment 27. And the depth of AMC has an influence on hydrothermal activity’s intensity and strength^29^. The hydrothermal sites are concentrated along the axis and at the western end of the neo-volcanic ridge (Fig. 1) while fewer hydrothermal plumes were observed on the eastern end of the ridge. Small low-velocity anomalies extending towards the western end of the segment provide evidence for magma redistribution at mid-crustal depth^23^. This magma provides sufficient heat source to the western end of the ridge axis and leads to the asymmetric plume distribution along the axis (Fig. 7). That may lead to the strongest CL-W hydrothermal field far away from the AMC centre.

At fast-spreading ridges, dyke leads to breaks in lithosphere and forms channels for upwelling of hydrothermal fluids^30^. Like this, dyke in segment 27 may provide channels for hydrothermal fluid(Fig. 7). The ORP-only vents suggest a crust with high ductility, few deep fractures, and mostly shallow, low-temperature hydrothermal circulation^3,10^ (Fig. 7).

With continued spreading, the off-axis lithosphere becomes cold and forms normal faults of varying sizes^31^. On ultraslow-spreading ridges, the heat source for off-axis hydrothermal circulation is magma away from the injection zone which is transported through the long-live boundary faults^31^. Faults can also act as pathways for the long-lasting circulation of high-temperature hydrothermal fluids that create vent fields.

## Conclusions

1. The hydrothermal activity along segment 27 is mainly characterized by wide-spread plumes. The plume heights are approximately 100–200 m and extend for ~3–10 km along the axis. Hydrothermal plumes are concentrated along the axial neo-volcanic ridge and at the western end of the ridge.
2. Based on the result of systematic hydrothermal exploration, particularly the use of ORP sensors, ridges can develop more vents than expected even on ultra-slow spreading ridges. Some previous studies have concluded that hotspot-affected ridges have less hydrothermal activity than other MORs with similar spreading rates. However, the hydrothermal activities at hotspot-affected ridges might be higher than previously thought in most locations that have not been systematically surveyed.
3. The AMC apparently provides sufficient heat source for wide-spread hydrothermal activity along segment 27. Dykes creates channels for heated fluids along the axis. Off-axis normal faults provide pathways for heat source to power flank vent fields.

## Methods

The methods used to detect hydrothermal activity along MORs can be broadly divided into large and small-scale investigations. One group is used for large-scale (tens to hundreds of kilometers) investigations that infer the source of hydrothermal vents^6^. For example, universal surveys use vertical CTD profiles at intervals of 10 km to obtain temperature, light scattering, and geochemical data in the water column^32,33^. Another type of investigation is conducted using a towed-line loaded with multiple sensors. One method is to use a tow-yo line with one sensor that traces a sawtooth path along the survey line, while another method uses several sensors loaded on a cable that traces fixed parallel paths and detects physical and chemical anomalies in the water column^34^. Small-scale investigations focus on precisely targeting hydrothermal vents using Autonomous Underwater Vehicles (AUVs) and Remote Operated Vehicles (ROVs) to identify hydrothermal activity by constructing high-resolution bathymetric maps of the seafloor, taking photographs, and finding water column anomalies from multiple sensors^35^.

DHDS was the main hydrothermal investigation instrument used during the 34^th^ *Dayang* cruise. The DHDS consisted of two parts. The first part comprised four to five Miniature Autonomous Plume Recorders (MAPRs) attached to a cable, which were placed at 50 m intervals above the bottom. The second part comprised multi-parameter chemical sensors (MPCS) and CTD sensors^36^, which were loaded onto the DHDS body. We also added a camera and video equipment to the DHDS body to obtain detailed images of the hydrothermal activity.

The MAPRs recorded light backscattering, temperature, and pressure data every 5 s. The raw voltage readings from the light backscattering sensors on the MAPRs are directly equivalent to Nephelometric Turbidity Units^8^. After processing the raw data, including the removal of random noise points, five-point moving averages were calculated and the background was subtracted. We obtained anomalous Nephelometric Turbidity Units values (ΔNTU) to discriminate high-temperature and particle-rich discharge^34^.

Oxidation-Reduction Potential (ORP, sometimes called Eh) data from MPCS can detect hydrothermal discharge close to the source (approximately ~1 km) at all temperatures, including low-temperature diffuse venting and higher-temperature yet particle-poor plumes^3^. The MPCS recorded ORP data every 5 s. Different ORP sensors have different background values, for data uniformity, we added the value of 200 mv to the original data. Because raw ORP data are readily influenced by electrode potential drifting^3,37^, the derivative of ORP to time dE/dt is calculated every 25 s (5 points).

Combined with ultra-short baseline (UBSL) position data, a camera and video are used to identify evidence of hydrothermal activity. Photos of seabed type anomalies such as reddish or brown hydrothermal sediments, sulfide deposit, and/or the presence of hydrothermal fauna are used to infer hydrothermal anomalies.

## Supplementary information

Supply information of manuscript

Sup. Table 1

Sup. Table 2

## Data Availability

The datasets generated and/or analyzed during the current study are available from the corresponding author upon reasonable request.

## References

[1] Baker, E. T. & German, C. R. On the Global Distribution of Hydrothermal Vent Fields. In *Mid-Ocean Ridges: Hydrothermal Interactions Between the Lithosphere and Oceans* 245–266 (2004).

[2] German CR, Petersen S, Hannington MD (2016). Hydrothermal exploration of mid-ocean ridges: Where might the largest sulfide deposits be forming?. Chem. Geol..

[3] Baker ET (2016). How many vent fields? New estimates of vent field populations on ocean ridges from precise mapping of hydrothermal discharge locations. Earth Planet. Sci. Lett..

[4] Hannington M, Jamieson J, Monecke T, Petersen S, Beaulieu S (2011). The abundance of seafloor massive sulfide deposits. Geology.

[5] Beaulieu SE, Baker ET, German CR (2015). Where are the undiscovered hydrothermal vents on oceanic spreading ridges?. Deep Sea Res. Part II Top. Stud. Oceanogr..

[6] Baker ET (2017). Exploring the ocean for hydrothermal venting: New techniques, new discoveries, new insights. Ore Geol. Rev..

[7] German CR (1998). Hydrothermal activity along the southwest Indian ridge.. Nature.

[8] Baker ET (2004). Hydrothermal venting in magma deserts: The ultraslow- spreading Gakkel and Southwest Indian Ridges. Geochemistry, Geophys. Geosystems.

[9] Devey CW, Lackschewitz KS, Baker E (2005). Hydrothermal and Volcanic Activity Found on the Southern Mid-Atlantic Ridge. EOS.

[10] Baker ET (2008). High-resolution surveys along the hot spot-affected Galápagos Spreading Center: 1. Distribution of hydrothermal activity. *Geochemistry*. Geophys. Geosystems.

[11] Dick HJB, Lin J, Schouten H (2003). An ultraslow-spreading class of ocean ridge. Nature.

[12] Ito, G., Lin, J. & Graham, D. Observational and theoretical studies of the dynamics of mantle plume-mid-ocean ridge interaction. *Rev. Geophys*. **41** (2003).

[13] Dyment J (1998). Evolution of the Carlsberg Ridge between 60 and 45 Ma: Ridge propagation, spreading asymmetry, and the Deccan‐Reunion hotspot. J. Geophys. Res. Solid Earth.

[14] Bruguier NJ, Minshull TA, Brozena JM (2003). Morphology and tectonics of the Mid-Atlantic Ridge, 7°–12°S. J. Geophys. Res. Solid Earth.

[15] Haase KM (2009). Diking, young volcanism and diffuse hydrothermal activity on the southern Mid-Atlantic Ridge: The Lilliput field at 9°33′S. Mar. Geol..

[16] Melchert B., Devey C.W., German C.R., Lackschewitz K.S., Seifert R., Walter M., Mertens C., Yoerger D.R., Baker E.T., Paulick H., Nakamura K. (2008). First evidence for high-temperature off-axis venting of deep crustal/mantle heat: The Nibelungen hydrothermal field, southern Mid-Atlantic Ridge. Earth and Planetary Science Letters.

[17] German CR (1994). Hydrothermal activity on the Reykjanes Ridge the Steinholl vent field at 63°06′N. Earth Planet. Sci. Lett..

[18] Haymon RM, White SM, Baker E (2008). High-resolution surveys along the hot spot–affected Gala´pagos Spreading Center: 3. Black smoker discoveries and the implications for geological controls on hydrothermal activity. *Geochemistry*. Geophys. Geosystems.

[19] Sauter D (2009). Propagation of a melting anomaly along the ultraslow Southwest Indian Ridge between 46°E and 52°20E: interaction with the Crozet hotspot?. Geophys. J. Int..

[20] Cannat M, Rommevaux-Jestin C, Sauter D, Deplus C, Mendel V (1999). Formation of the axial relief at the very slow spreading Southwest Indian Ridge (49° to 69°E). J. Geophys. Res..

[21] Mendel V., Sauter D., Rommevaux-Jestin C., Patriat P., Lefebvre F., Parson L. M. (2003). Magmato-tectonic cyclicity at the ultra-slow spreading Southwest Indian Ridge: Evidence from variations of axial volcanic ridge morphology and abyssal hills pattern. Geochemistry, Geophysics, Geosystems.

[22] Jian, H., Singh, S. C., Chen, Y. J. & Li, J. Evidence of an axial magma chamber beneath the ultraslow- spreading Southwest Indian Ridge. **44**, 2–5 (2016).

[23] Jian H (2017). Seismic structure and magmatic construction of crust at the ultraslow-spreading Southwest Indian Ridge at 50°28′E. J. Geophys. Res. Solid Earth.

[24] Yang AY, Zhao TP, Zhou MF, Deng XG (2017). Isotopically enriched N-MORB: A new geochemical signature of off-axis plume-ridge interaction—A case study at 50°28′E, Southwest Indian Ridge. J. Geophys. Res. Solid Earth.

[25] Tao C (2012). First active hydrothermal vents on an ultraslow-spreading center: Southwest Indian Ridge. Geology.

[26] Baker, E. T., German, C. R. & Elderfield, H. Hydrothermal plumes over spreading‐center axes: Global distributions and geological inferences. In *Seafloor Hydrothermal Systems: Physical, Chemical, Biological, and Geological Interactions* 47–71 (1995).

[27] Liao S (2018). Bulk geochemistry, sulfur isotope characteristics of the Yuhuang-1 hydrothermal field on the ultraslow-spreading Southwest Indian Ridge. Ore Geol. Rev..

[28] Zhang T, Lin J, Gao JY (2013). Magmatism and tectonic processes in Area A hydrothermal vent on the Southwest Indian Ridge. Sci. China:Earth Sci..

[29] Baker ET (2009). Relationships between hydrothermal activity and axial magma chamber distribution, depth, and melt content. Geochemistry, Geophys. Geosystems.

[30] German, C. R. & Lin, J. The Thermal Structure of the Oceanic Crust, Ridge-Spreading and Hydrothermal Circulation: How Well Do We Understand Their Inter-Connections? In *Mid-Ocean Ridges: Hydrothermal Interactions Between the Lithosphere and Oceans* 1–18 (2004).

[31] Standish JJ, Sims KWW (2010). Young off-axis volcanism along the ultraslow-spreading Southwest Indian Ridge. Nat. Geosci..

[32] Edmonds HN (2003). Discovery of abundant hydrothermal venting on the ultraslow-spreading Gakkel ridge in the Arctic Ocean. Nature.

[33] Kawagucci S (2008). Methane, manganese, and helium-3 in newly discovered hydrothermal plumes over the Central Indian Ridge, 18°-20°S. *Geochemistry*. Geophys. Geosystems.

[34] Walker SL, Baker ET, Massoth GJ, Richard HN (2004). Short-term variations in the distribution of hydrothermal plumes along a superfast spreading center, East Pacific Rise, 27°30′–32°20′S. Geochemistry, Geophys. Geosystems.

[35] German CR (2008). Hydrothermal exploration with the Autonomous Benthic. Explorer. Deep. Res. Part I Oceanogr. Res. Pap..

[36] Chen S (2014). A data processing method for MAPR hydrothermal plume turbidity data and its application in the Precious Stone Mountain hydrothermal field. Acta Oceanol. Sin..

[37] Baker ET (2014). Correlated patterns in hydrothermal plume distribution and apparent magmatic budget along 2500 km of the Southeast Indian Ridge. Geochemistry, Geophys. Geosystems.

[38] Li J (2015). Seismic observation of an extremely magmatic accretion at the ultraslow spreading Southwest Indian Ridge. Geophys. Res. Lett..

