# Supplementary material for: Seafloor hydrothermal activity along mid-ocean ridge with strong melt supply: study from segment 27, southwest Indian ridge
Source: Sci Rep. 2019 Jul 8;9:9874. doi: 10.1038/s41598-019-46299-1 (PMC6614410; doi:10.1038/s41598-019-46299-1)
Supplement: Supplementary file 1 — Supply information of manuscript [file 41598_2019_46299_MOESM1_ESM.docx]

**Seafloor hydrothermal activity along mid-ocean ridge with strong melt supply: study from segment 27, southwest Indian ridge**

Xihe Yue^1,2^, Huaiming Li^2^*, Jianye Ren^1^, Chunhui Tao^2,3^, Jianping Zhou^2^, Yuan Wang^2^, Xiaoxia Lü^1^

^1^ College of Marine Science and Technology, China University of Geosciences, Wuhan, 430074, China

^2^ Key Laboratory of Submarine Geosciences, State Oceanic Administration, Second Institute of Oceanography, Ministry of Natural Resources, Hangzhou, 310012, China

^3^ Institute of Oceanography, Shanghai Jiao Tong University, Shanghai, 200030, China

* Corresponding Authors: [huaiming_lee@163.com](mailto:huaiming_lee@163.com)


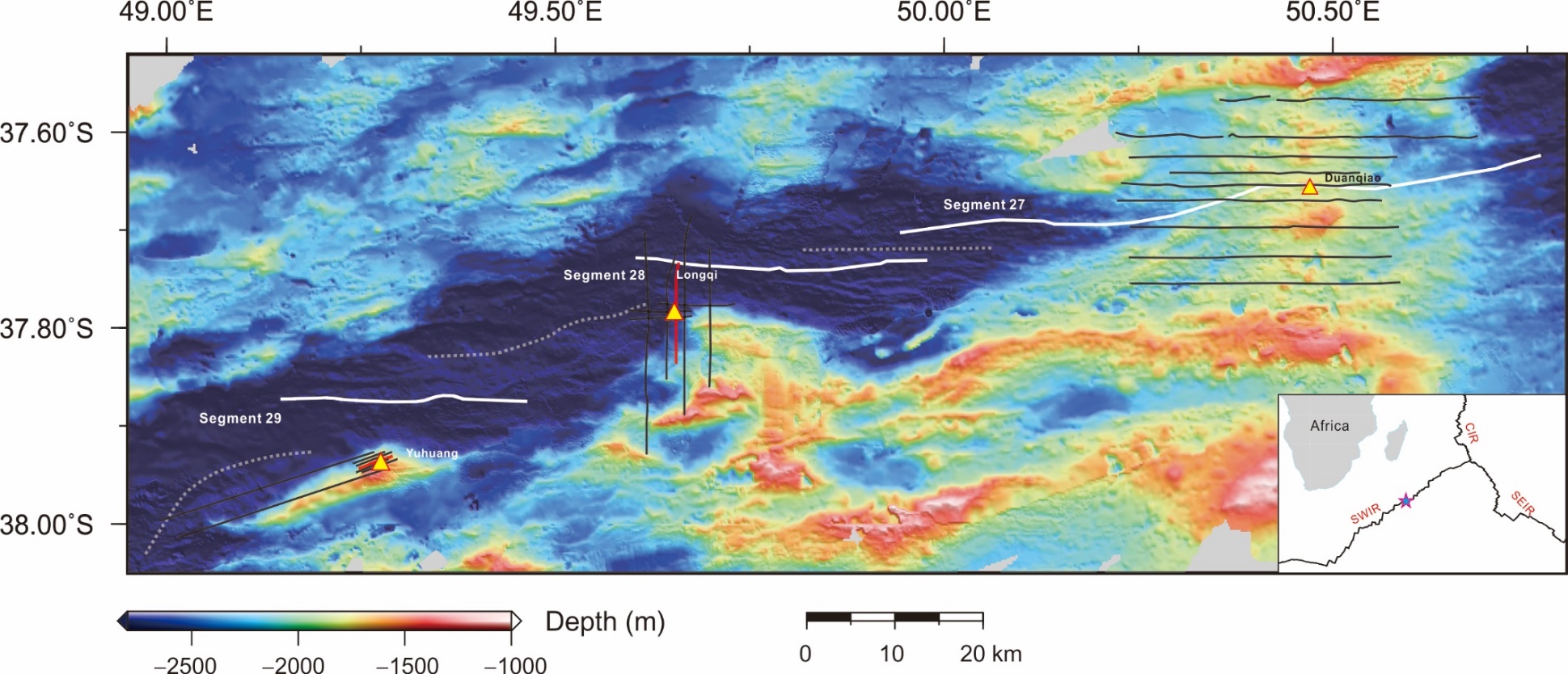


Sup. Figure 1 The location of segment 27-29. (Created by the Generic Mapping Tools (GMT version 5), from http://gmt.soest.hawaii.edu/. The topography is from multibeam sonar data by Chinese Dayang cruises). The black and red lines show the DHDS survey lines of the 34^th^ Dayang cruise. The line in Figure 2 is in red. The white lines indicate the axial volcanic ridge (AVR). The white dotted lines indicate the non-transform discontinuities (NTDs) (Cannat et al., 1999, Sauter et al.,2001). The red triangles denote the hydrothermal fields . The right inset shows the location of the study area(Created by the Generic Mapping Tools (GMT version 5). Ridge data from: http://www-udc.ig.utexas.edu .SWIR: Southwest Indian Ridge; CIR: Central Indian Ridge; SEIR: Southeast Indian Ridge.

Sup. Figures of survey lines

| 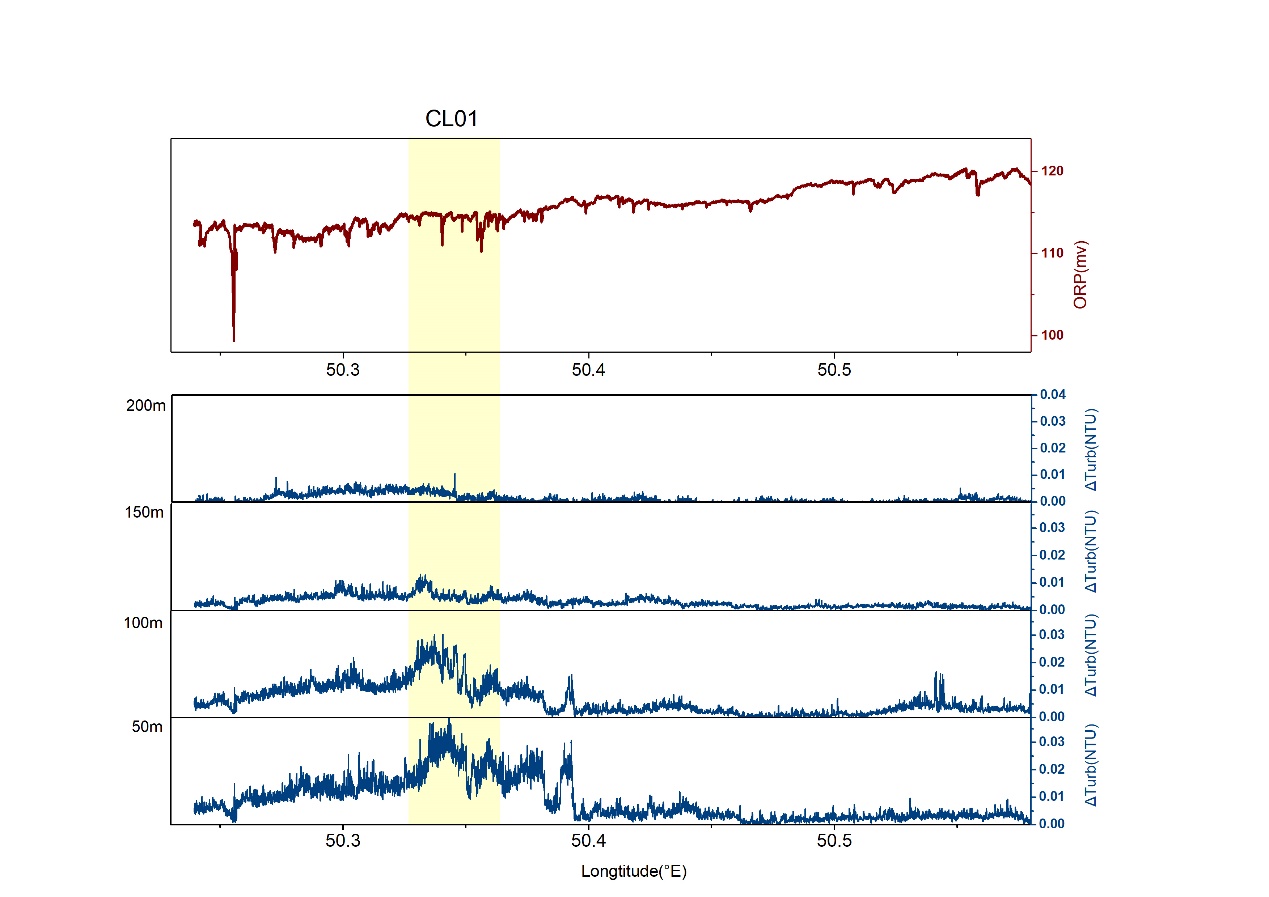 |
| --- |
| Sup. Figure 2 The CL01 line ORP and individual MAPRs data from 50-200m altitudes. |
| 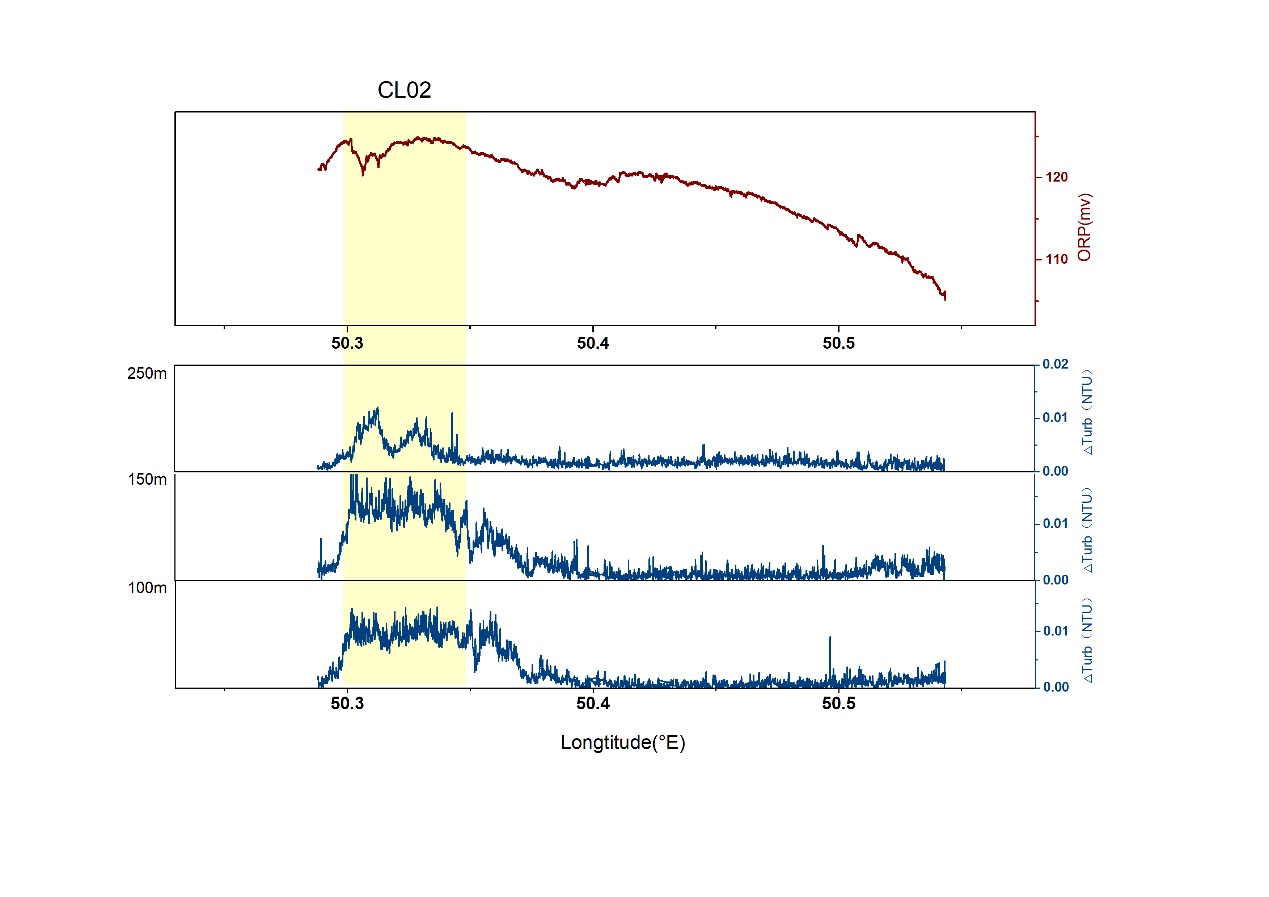 |
| Sup. Figure 3 The CL02 line ORP and individual MAPRs data from 100-250m altitudes. |
| 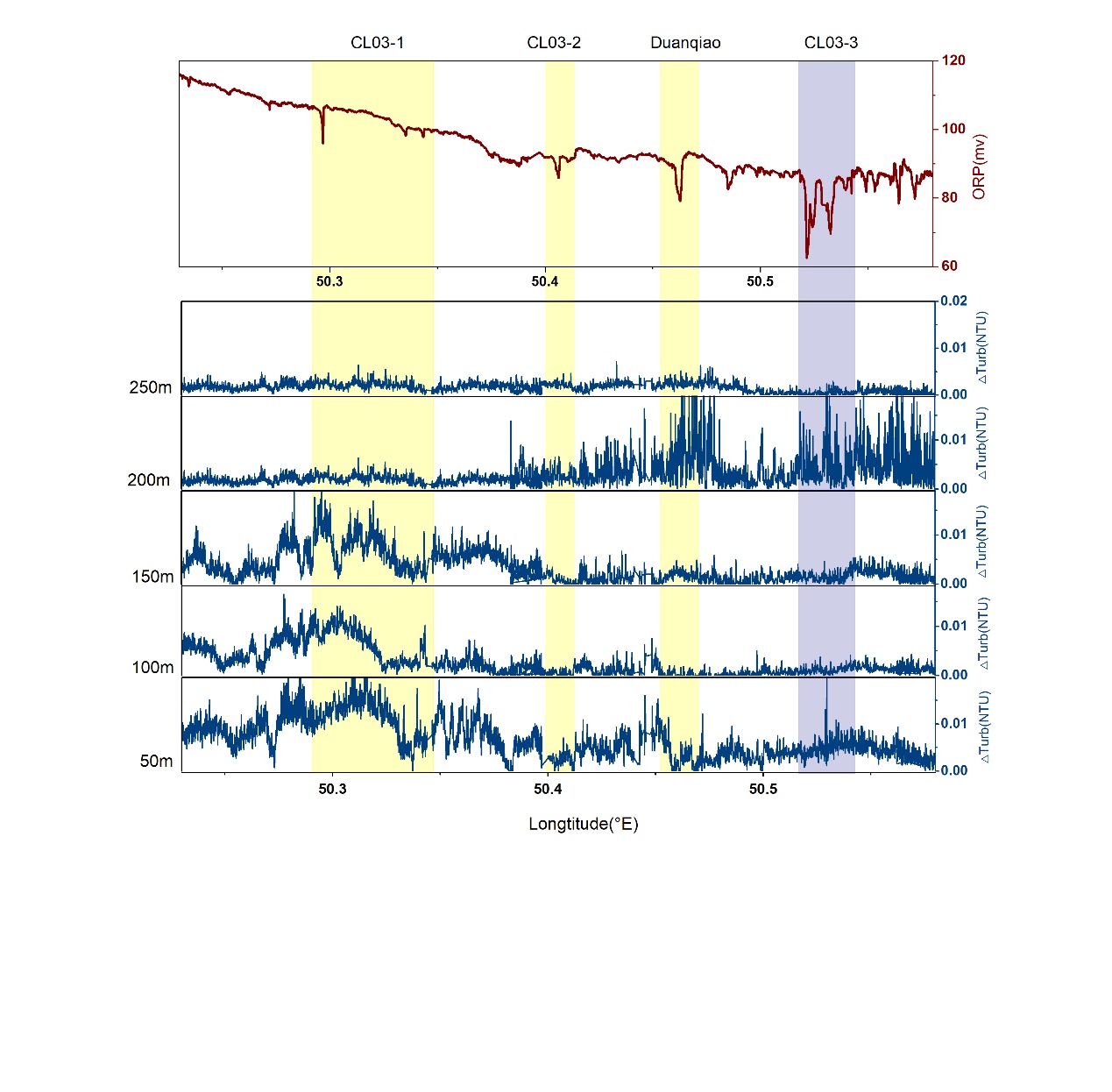 |
| Sup. Figure 4 The CL03 line ORP and individual MAPRs data from 50-250m altitudes. |
| 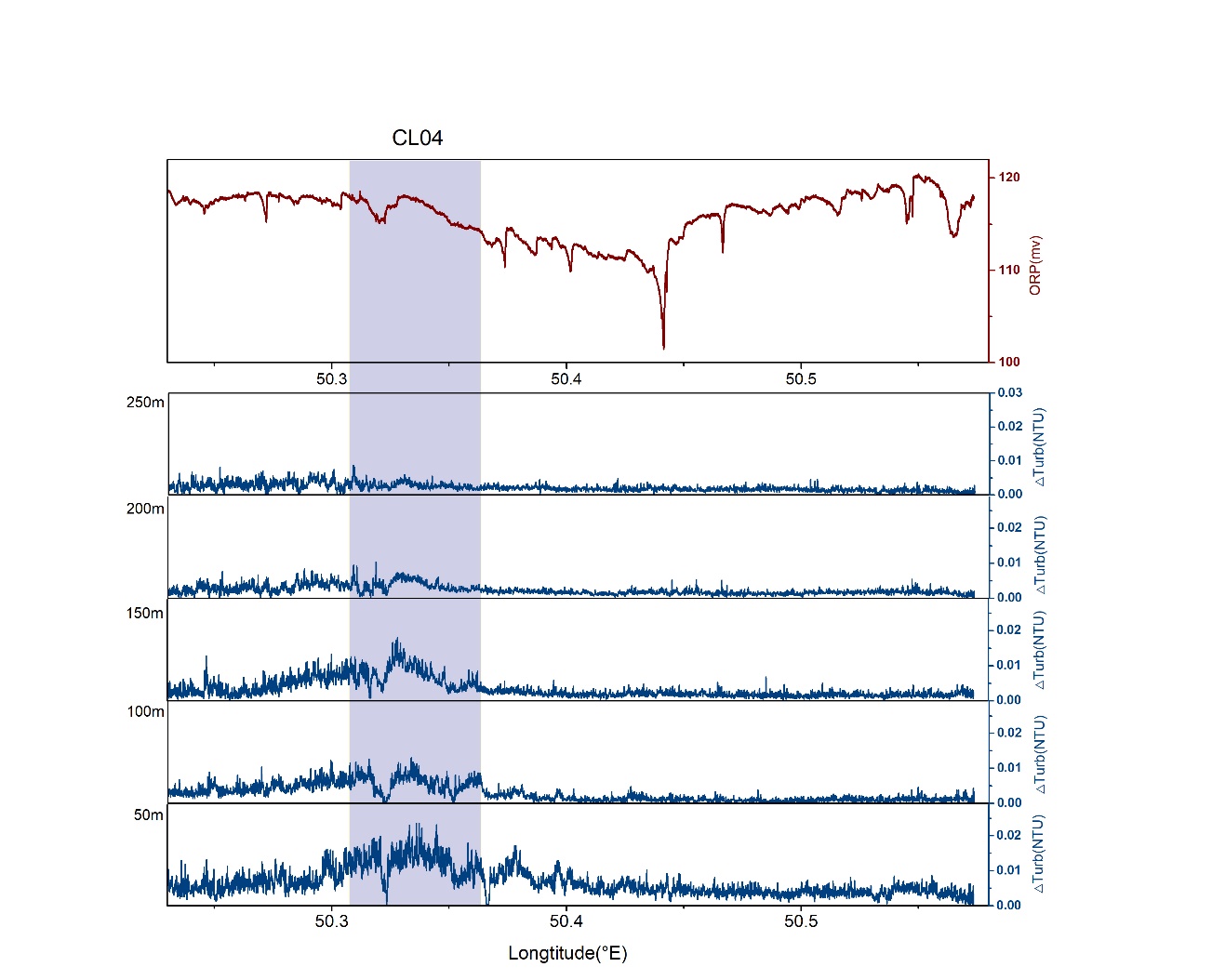 |
| Sup. Figure 5 The CL04 line ORP and individual MAPRs data from 50-250m altitudes. |

| 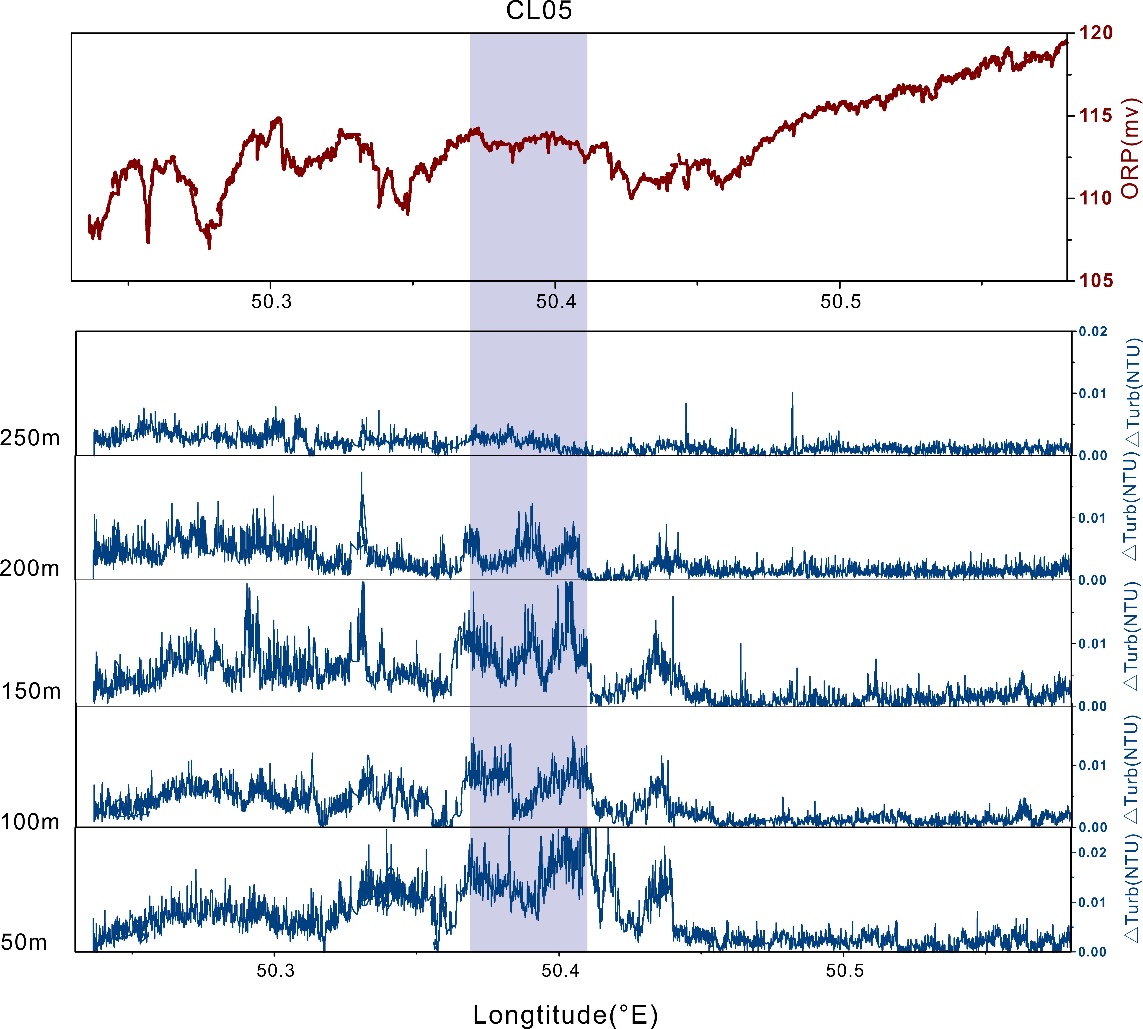 |
| --- |
| Sup. Figure 6 The CL05 line ORP and individual MAPRs data from 50-250m altitudes. |

| 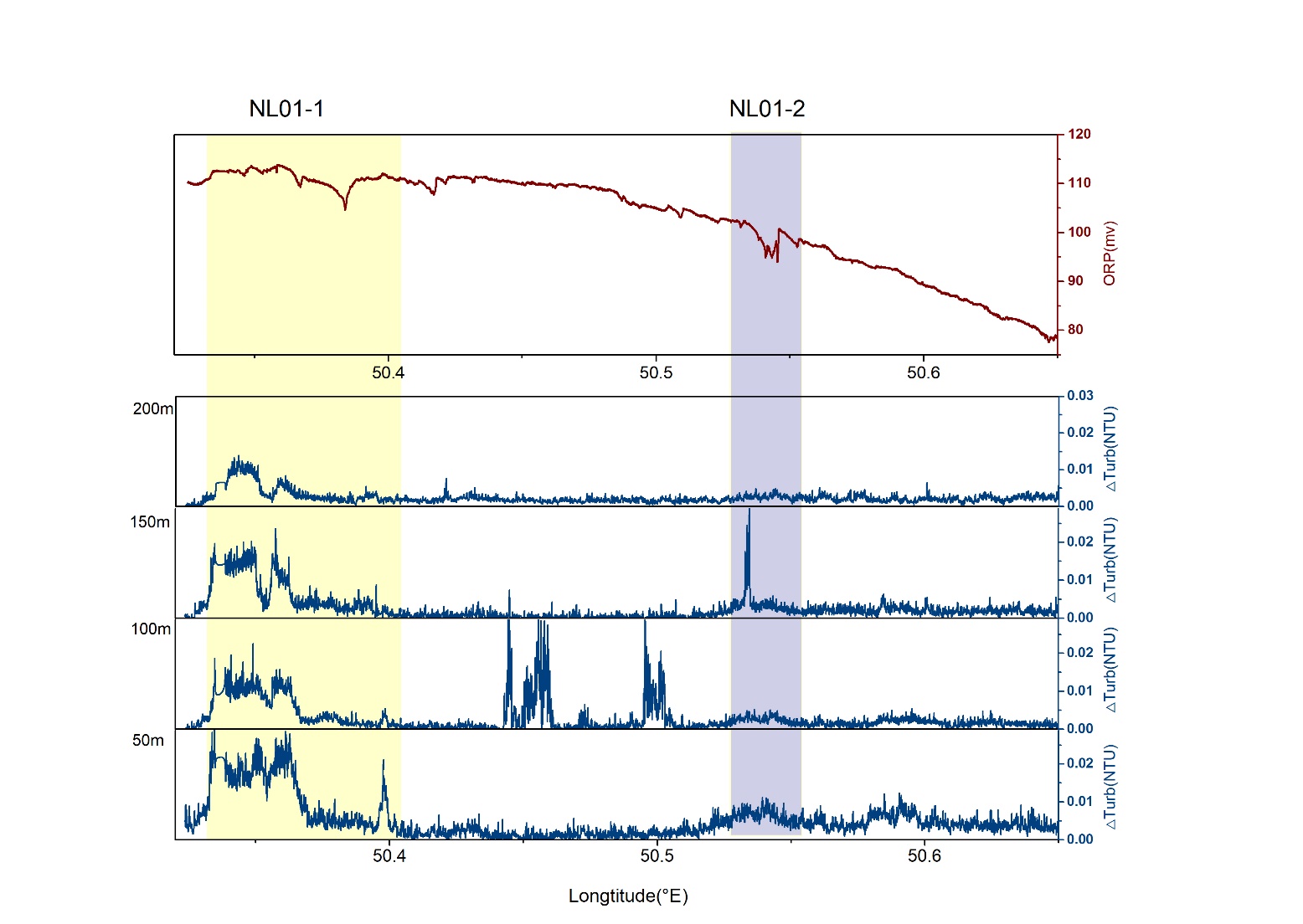 |
| --- |
| Sup. Figure 7 The NL01 line ORP and individual MAPRs data from 50-200m altitudes. |
| 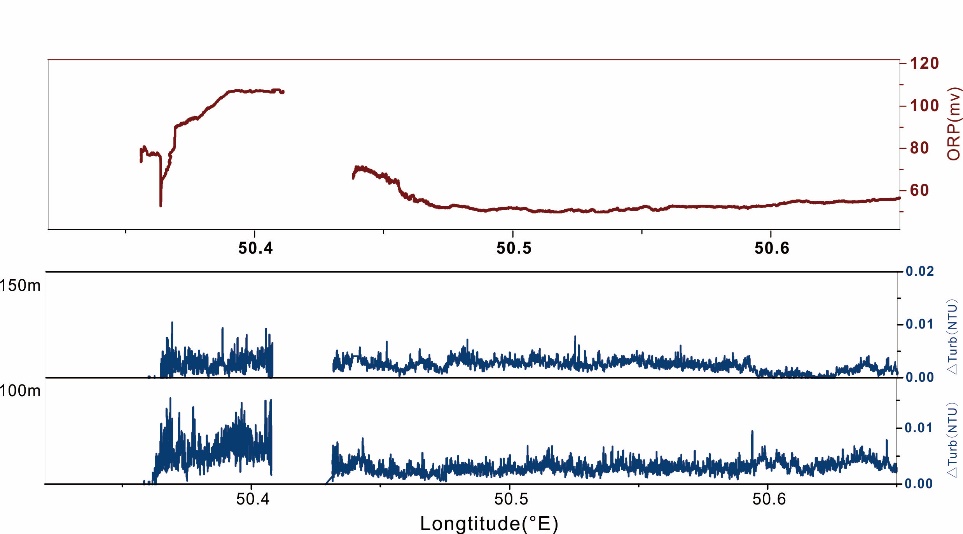 |
| Sup. Figure 8 The NL02 line ORP and individual MAPRs data from 100-150m altitudes. |

| 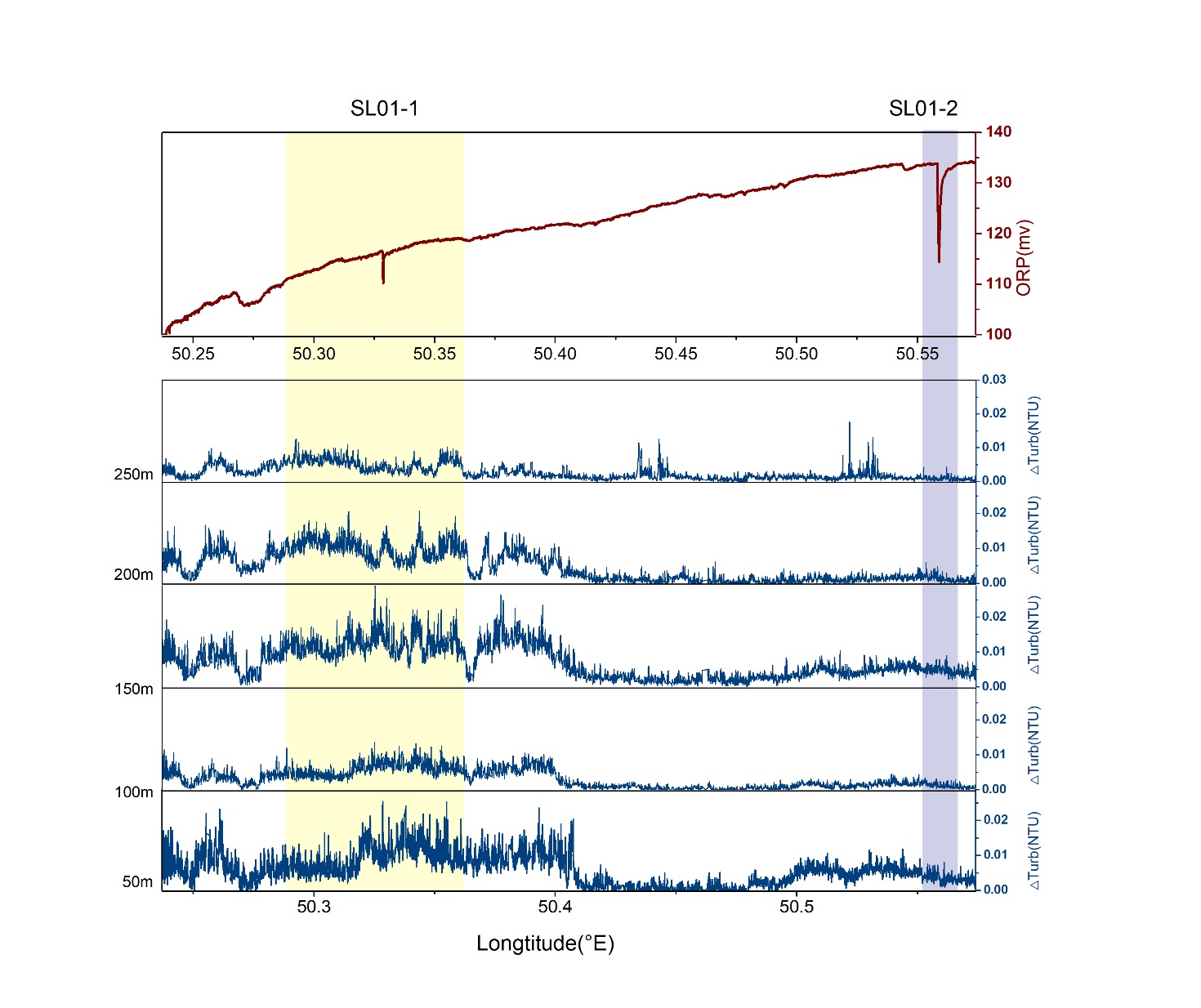 |
| --- |
| Sup. Figure 9 The SL01 line ORP and individual MAPRs data from 50-250m altitudes. |
| 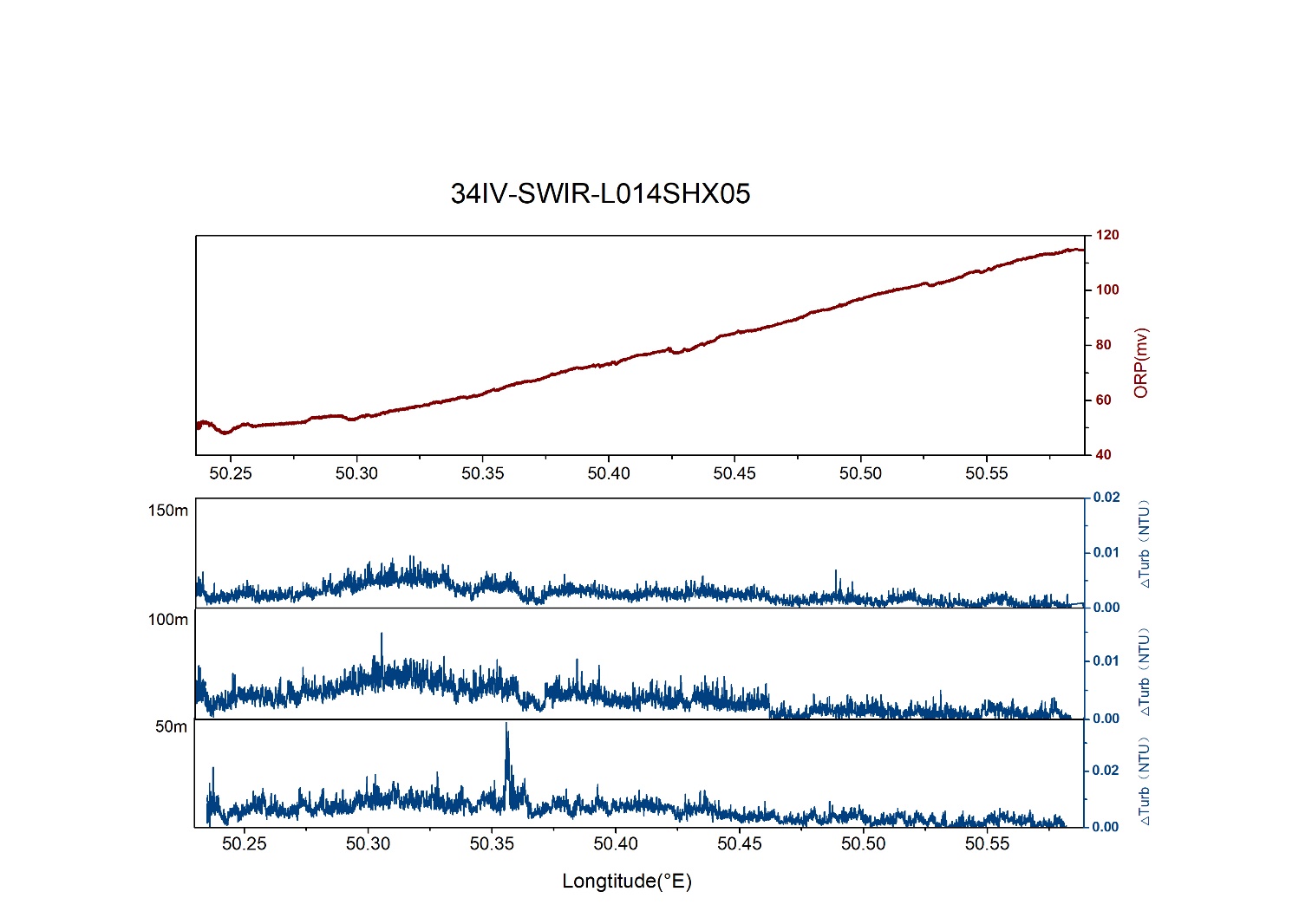 |
| Sup. Figure 10 The SL02 line ORP and individual MAPRs data from 50-250m altitudes. |
